# Effects of inflammation on myopia: evidence and potential mechanisms

**DOI:** 10.3389/fimmu.2023.1260592

**Published:** 2023-10-02

**Authors:** Ran Xu, Jing Zheng, Longqian Liu, Wenqiu Zhang

**Affiliations:** ^1^ Department of Ophthalmology, West China Hospital, Sichuan University, Chengdu, China; ^2^ Department of Optometry and Visual Science, West China Hospital, Sichuan University, Chengdu, China

**Keywords:** myopia, inflammation, anti-inflammatory drugs, immune diseases, scleral remodeling

## Abstract

As the most common type of refractive error, myopia has become one of the leading causes of visual impairment. With the increasing prevalence of myopia, there is a growing need to better understand the factors involved in its development. Inflammation, one of the most fundamental pathophysiological processes in humans, is a rapid response triggered by harmful stimuli and conditions. Although controlled inflammatory responses are necessary, over-activated inflammation is the common soil for many diseases. The impact of inflammation on myopia has received rising attention in recent years. Elevated inflammation may contribute to myopia progression either directly or indirectly by inducing scleral remodeling, and myopia development may also increase ocular inflammation. This article provides a comprehensive review of the interplay between inflammation and myopia and the potential biological mechanisms, which may present new targets for understanding the pathology of myopia and developing myopia therapies.

## 1 Introduction

When ocular accommodation is relaxed, light entering the eye from the external environment parallel to the optical axis is focused in front of the retina, which is called myopia. During the past 30 years, the myopia population has increased rapidly worldwide, especially in East and Southeast Asia (1, 2), with the prevalence of myopia among 16-18-year-olds even reaching 84.8% in China (3). It has been projected that by 2050, half of the total global population will be myopic (4).Uncorrected refractive error and pathological myopia have become major causes of visual impairment and even blindness (5, 6). Therefore, it is particularly important to prevent or delay myopia onset.

A series of signaling pathways, including dopamine (7), retinoic acid (8), Wnt/β-catenin (9), transforming growth factor-β (TGF-β) (10), and hypoxia-inducible factor-1 alpha (HIF-1α) (11) signaling pathways, have been confirmed to be associated with the development of myopia; however, the exact pathogenesis of myopia remains unclear since myopia is regulated by both genetic and environmental factors (12).

Inflammation is an adaptive response of the body to harmful stimuli and is beneficial under controlled conditions (13). As one of the few organs in the body with immune privilege, the eye has unique immunological properties. In general, the ocular microenvironment is both immunosuppressive and anti-inflammatory (14). Under physiological conditions, various endothelial cells, immune cells (such as microglia, neutrophils, monocytes, macrophages, natural killer cells, etc.) and retinal neuronal cells inhibit the activity of effector T cells through the release of immunosuppressive factors, promote the formation and maintenance of ocular immune privilege and prevent ocular excessive inflammation (15). Whereas when the eye is subjected to sustained stimulation of damage-associated molecular patterns, local chronic inflammation mediated by innate immune cells develops (16), ultimately leading to destructive tissue remodeling and loss of visual function through a series of cascade reactions. It has been shown that a low-grade chronic inflammatory response in the eye is associated with the decreased function of retinal pigment epithelial (RPE), breach of the blood-retinal barrier, neovascularization and choroidal macrophages recruitment (17). Although inflammation has been identified in relation to many ocular diseases, the association between it and myopia has not been confirmed. Recently, researchers have turned their attention to this area, offering new insights into the specific mechanisms of myopia. Inflammatory cytokines activated by mitogen-activated protein kinase (MAPK), nuclear factor kappa B (NF-κB) and other signaling pathways transmit from the retina to the sclera (18), then directly or indirectly promote myopia.

This article reviews the evidence and potential mechanisms linking inflammation and myopia, and describes the specific association between myopia and inflammatory or immune diseases. In addition, it discusses the potential of anti-inflammatory products in the treatment of myopia.

## 2 Clinical evidence linking inflammation and myopia

Increasing clinical evidence has shown that the higher inflammatory status in circulation system or within the eye indicates the severity of myopia and that inflammation may function in the pathogenesis of myopia.

### 2.1 Systemic inflammatory status and myopia

Recent studies have found that the systemic immune system is involved in myopia. Elevated certain systemic markers suggesting inflammation and imbalances in circulating immune cells in patients with high myopia or pathological myopia indicated that myopic patients might have a systemic hypo-inflammatory status.

Data from a large cross-sectional study (19) in Korea showed that higher white blood cell counts were significantly associated with increased myopia prevalence. Meanwhile, elevated circulating neutrophils as well as decreased monocytes, eosinophils, and lymphocytes were found during the progression of myopia (20). As indicators of systemic inflammatory properties (21), significantly higher neutrophil-to-lymphocyte ratios and platelet-to-lymphocyte ratios in the peripheral blood of patients with high myopia (22, 23) also implied higher inflammation and dysregulation of serum immune cells in high myopia population than in normal subjects. Significant elevations in high-sensitivity C-reactive protein and the complement profile in the peripheral blood of patients with pathological myopia (24) suggested the presence of systemic immune microinflammation in pathological myopia. In addition, Dai et al (25) elaborated the relevance of oxidative stress, inflammation and metabolic changes in high myopia in a study of human serum metabolomics.

However, such studies have not demonstrated a correlation between systemic inflammatory levels and the eye. Besides, they might not exclude mixing effects of underlying systemic inflammatory disease.

### 2.2 Inflammatory status in the ocular microenvironment of myopia

Current studies tend to consider that the visual mechanisms regulating refractive development are located primarily in the retina (26). Hence, the formation of myopia is considered as a localized stimulatory process, and the correlation between myopia and inflammation of the ocular environment has received more attention than systemic status.

Several studies (27–30) have shown that compared to non-myopic eyes, myopic eyes have higher levels of inflammatory cytokines in the aqueous humor or vitreous. The levels of interleukin (IL)-6 and metalloproteinase-2 (MMP-2) in aqueous humor were positively correlated with the ocular axial length (AL), and they were also significantly higher in highly myopic eyes than in control eyes (27). The expression of monocyte chemoattractant protein-1 (MCP-1) in the aqueous humor was significantly higher in highly myopic cataract patients than in age-related cataract patients, whereas the expression of IL-1 receptor antagonists was significantly lower (28). In addition, the expression of inflammatory cytokines, such as interferon gamma (IFN-γ), IL-6, interferon-inducible protein 10, eotaxin, MCP-1, macrophage inflammatory protein-1α (MIP-1α), and MIP-1β, was also elevated in the vitreous of patients with high myopia (29) as well as in highly myopic eyes with macular holes (MHs) (30). Raised MIP originating from macrophages, dendritic cells and lymphocytes implies that immune cells participate in myopia formation.

Studies of bioinformatics analysis likewise confirmed that interactions of complement activation, immunity and inflammation, and extracellular matrix remodeling may play a role in the pathogenesis of myopia, especially pathologic myopia (30–32). Differentially expressed miRNAs in vitreous revealed that several signaling pathways, such as the MAPK, phosphatidylinositol 3-kinase (PI3K)/protein kinase B (AKT), T-cell receptor and chemokine signaling pathways associated with inflammation, were enriched in highly myopic MH eyes (30). Imbalance of the MAPK signaling pathway may also be one of the key steps of lens alteration in highly myopic eyes (31). Analysis of gene expression profiles in the cornea showed that immune-related pathways were significantly enriched in myopia. Meanwhile, infiltrating immune cell analysis of the myopic cornea revealed significant enrichment of B cells, CD4+ memory T cells, CD8+ central memory T cells, T helper 2 (Th2) cells, regulatory T cells (Tregs), etc. whereas CD8+ T cells, CD4+ T central memory cells and T helper 1 (Th1) cells were reduced (33).

The elevated inflammatory cytokines prove the presence of higher-than-normal ocular inflammatory status in myopic eyes, suggesting that the continuous subclinical inflammation within the eye may lead to myopia progression. However, there was an inconsistent report on the association between myopia and inflammation. Zhu et al (34) showed no significant link between AL and inflammatory cytokines (IL-1β, IL-6 and tumor necrosis factor-alpha (TNF-α)) in aqueous humor in cataract patients with AL ranging from 22.6-31.5 mm. The conflicting results could be due to differences in sample size, testing instruments, and inclusion criteria.

It should be noted that current clinical evidence cannot fully elucidate the correlation between myopia and inflammation. First, the results are not representative, because the aqueous humor and vitreous samples involved in these studies could only be obtained surgically. The level of inflammation in myopic patients who do not require surgery is not known. Second, the characteristics of subjects in myopia stabilization and the design of cross-sectional studies limit the determination of the causal relationship between myopia and inflammation. Besides, pathologic myopia itself has been shown to be related to the autoimmune and inflammatory systems (35). Therefore, the basic experiments are needed to provide supporting evidence for previous clinical contribution.

## 3 Inflammation in experimental myopic model

Studies have shown that expression of ocular inflammatory cytokines increases with the development of myopia in different species of experimental myopia, including hamsters (18, 36–38), tree shrews (39), mice (40), guinea pigs (41, 42), and chicks (43), and that increased ocular inflammatory status promotes the development of myopia.

The inflammatory cytokines were activated and transmitted from retinal to scleral during myopia induction. The inflammation-associated transcription factors c-Fos and NF-κB and the inflammatory cytokines IL-6, TNF-α, IL-1β, TGF-β and MMP-2 were upregulated, while the anti-inflammatory cytokine IL-10 was decreased in myopic eyes of Syrian hamsters induced by form-deprivation myopia (FDM) (18), suggesting that pathways associated with inflammation-induced myopia may include the MAPK and NF-κB pathways. Meanwhile, the immunosuppressive agent cyclosporine A applied to the eye delayed myopia progression, while inflammatory stimulators peptidoglycan and lipopolysaccharide promoted myopia progression (18). Several other studies (36, 37) likewise observed that inflammation-related factors increased in the FDM eye of hamsters with myopia progression and that the application of pro-inflammatory agents could also promote the expression of these factors in RPE cells *in vitro*. RPE cells play an important role in myopigenesis, which can recognize key signaling molecules and influence ion and fluid transport to transmit growth-regulating signals from retina to choroid/sclera (44). Similar experimental results were demonstrated in tree shrews undergoing 7/14-day FDM (39).

Since the main innate inflammatory cell population that has been described in ocular inflammation is macrophages (16), the investigators discussed the link between macrophages and myopia. It was found that in addition to scleral fibroblasts, monocyte-derived scleral macrophages induced by scleral C-C motif chemokine ligand-2 (CCL2) were one of the sources of MMP-2 in the eye (45). Secreted MMP-2 hydrolyzed collagen fibrils and recollects monocytes and neutrophils (46). In cases of negative lens-induced myopia, macrophage-like cells were observed to directly phagocytose collagen fibrils as well as fibroblasts in sclera (47). This indicates that macrophages contribute to the development of myopia.

In addition, RNA sequencing results also demonstrated the activation of inflammation-related signaling pathways in experimental myopic eyes. Single-cell RNA sequencing showed sustained expression of MAPK, and PI3K/AKT signaling pathways in scleral fibroblasts from mice experiencing 4 weeks of FDM (40). RNA-seq analysis of retinas from guinea pigs undergoing 15 weeks of FDM (41) suggested that inflammatory pathways such as inflammatory mediator regulation of Tryptophan channels and IL-17 signaling pathway played crucial roles in of myopia-induced retinal degeneration.

The complement system is an important component of innate immunity (48). The level of C5b-9 was significantly elevated in the posterior sclera of guinea pigs (42) with negative lens-induced myopia (LIM), with increased C1q and C3 protein expression. Transcription and activation of the complement system were also present during the induction of myopia and hyperopia in chicks (43).Activation of the complement system may trigger inflammatory responses in some ocular diseases such as primary angle closure glaucoma (49), and these inspire us that complement may function in myopia through inflammation.

In contrast to previous studies (18), Jody et al (50) argued that the recovery from FDM or myopic defocus affecting the expression of those proinflammatory cytokine, despite affirming that IL-6 in the choroid plays an important role in the retina-sclera signaling cascade. They found that IL-6 expression was increased in the chick choroid during recovery from FDM or during the application of a +15D lens and was upregulated in myopic eyes treated with atropine. However, unlike the previous experimental design, this study focused on IL-6 in the choroid.

In conclusion, most studies agree that the development of myopia is accompanied by increased levels of ocular inflammation, and that increased inflammation in the eye predisposes to myopia.

## 4 Correlation between myopia and inflammatory or immune diseases

Several studies have noted a link between inflammatory or immune diseases and myopia, with patients who exist abnormal inflammatory or immune status having a higher prevalence of myopia than normal individuals. These diseases may be a risk factor for myopia, although the correlation has not been fully evaluated.

### 4.1 Ocular diseases

It has been reported that ocular inflammatory diseases, including allergic conjunctivitis (AC) (51, 52), scleritis (53–55), and uveitis (18, 56, 57), may trigger the progression of myopia by elevating ocular inflammation.

A study by Mimura (51) showed that AC patients who were positive for specific immunoglobulin E to indoor allergens exhibited higher degree of myopia than healthy subjects, linking allergic conjunctivitis to myopia for the first time. Subsequently, a case−control and cohort study (52) showed a significantly higher risk and incidence of myopia in patients with AC than in those with nonallergic conjunctivitis, validating the causal relationship between AC and myopia. Furthermore, they also demonstrated again in Lewis rat model of AC that the presence of AC triggers ocular surface inflammation, activates pro-inflammatory factors, leading to scleral remodeling and eye axis growth (52).

The association between scleritis and myopia has mainly been focused on case reports. Fan (53) and Ugurbas (54) reported that in addition to common ocular pain, scleritis may occur as sudden monocular myopia, followed by typical clinical and imaging findings. During follow-up, 10-30% of patients with necrotizing scleritis may have a visual acuity of 0.1 or worse at some point in time (55). Although myopia is not a prevalent symptom of scleritis, there seems to be an association between them.

Uveitis can cause acute, transient or persistent myopia in different conditions (56). A retrospective cohort study (18) found an increased risk and cumulative incidence of myopia in patients with uveitis. Meanwhile, data from research (57) with a 15-year follow-up of chorioretinal inflammatory diseases showed that myopic refractive changes were present in some inflammatory disease, including multifocal choroiditis and panuveitis (mean -2.19 D), punctate inner choroidopathy (mean -3.67 D), diffuse subretinal fibrosis syndrome (mean -1.25 D), and multiple evanescent white dot syndrome (mean -1.25 D).

The high risk of myopia due to AC may be associated with elevated ocular surface inflammation, whereas the cause of myopia in patients with scleritis and uveitis is more often thought to be changes in lens refractive power and the elongation of AL. Inflammation of the sclera and uvea may present with uveal effusion, ciliary exudation, swelling, or detachment, leading to relaxation of the suspensory ligaments and zonular fibers, increasing the refractive power of the lens and causing myopic shift (53). In addition, elevated inflammatory cytokines such as IL-1 and TNF-α in uveitis (58) and scleritis (59) may lead to the dysregulation of MMPs and tissue inhibitors of metalloproteinases (TIMPs), resulting in degradation of scleral collagen, and the reduction in perfusion of ocular vasculature caused by inflammation (60, 61) may likewise lead to scleral ischemia, hypoxia and remodeling.

### 4.2 Systemic diseases

The relationship between myopia and systemic immune or inflammatory diseases has gradually attracted researchers’ attention. Diseases such as Vogt−Koyanagi−Harada (VKH) disease, juvenile idiopathic arthritis (JIA), systemic lupus erythematosus (SLE), and Kawasaki disease (KD) were reported to be associated with myopia.

VKH disease is a T-cell-mediated systemic autoimmune disease that primarily targets melanocytes (62). A retrospective study (63) about refractive changes in VKH patients found that 16% of eyes showed significant myopic progression and that sunset glow fundus was more frequent in these patients than in those without myopia progression. Some patients with chronic VKH also experienced myopia progression and growth of AL (64). JIA is an autoinflammatory disease, which is a general term covering all arthritis of unknown origin that develops under the age of 16 years and lasts for more than 6 weeks (65). A previous study (66) reported that the prevalence of myopia was increased in patients with juvenile chronic arthritis compared to their peers and that myopia occurred after the diagnosis of this disease. The results of another research (67) also found 42% of 40 JIA patients had myopia, but the number of myopic patients was not significantly different between those with and without uveitis. Thus, the elevated risk of myopia in patients with JIA may not completely be associated with induced uveitis. The mechanism of their induction of myopia is more likely to be through the increased inflammatory status of the eye caused by the diseases themselves.

SLE is a chronic, autoimmune inflammatory connective tissue disease affecting multiple organ systems. Although myopia is not a common ocular manifestation of SLE, several cases have reported that acute episodes of reversible myopia may be a feature of SLE (68–70). A retrospective study (18) likewise showed that patients with SLE had a higher risk of myopia and a higher cumulative prevalence of myopia than healthy patients of the same age. Similar to scleritis and uveitis, the pathogenesis of myopia due to SLE may lie in uveal effusion and ciliary swelling due to choroidal vasculitis (71), which subsequently cause lens-induced myopic shift. When the inflammatory response subsides or is controlled, part of the myopic shift may gradually disappear.

KD is a systemic inflammatory disease of unknown etiology, where one of its pathogenesis lies in dysregulation of the immune system and abnormal T-cell function due to triggering of the inflammatory cascade response (72). A prospective cohort study (73) of KD and non-KD children aged 0-6 years in Taiwan found that KD was an independent risk factor for myopia regardless of age, sex, and urbanization. And the risk of myopia in KD patients increased with the growth of age as well as frequency of clinic visits. The incidence of myopia was significantly lower in KD patients treated with intravenous immunoglobulin (IVIG) than in those treated with aspirin alone (74). Although both aspirin and immunoglobulin have anti-inflammatory effects, the anti-inflammatory effect of IVIG appears to be stronger than that of aspirin, with patients receiving only aspirin or low IVIG + aspirin having a significantly longer duration of fever and a significantly higher incidence of coronary aneurysms than KD patients receiving high IVIG + aspirin (75). Thus, the lower risk of myopia in patients receiving IVIG could be explained from the perspective of reducing the inflammatory response, and the association between myopia and inflammatory diseases could also be supported.

In summary, the evidence described above implied that myopia was related to the inflammatory features of certain diseases. However, most of these studies were cross-sectional without showing specific causal relationships, and studies correlating refractive error or AL with the severity of inflammation are lacking. It should be noted that due to restricted physical ability, patients with chronic inflammatory diseases may spend less time outdoors or expose to the natural light, and may also experience more time in close proximity or using electronics, all of above are risk factors for myopia. In addition, the refractive power of the eye depends on the curvature of surface of cornea and lens, refractive indices of refractive medium and AL (76), all of which are not clearly related to inflammation. Therefore, there is still insufficient evidence to determine whether inflammation is an independent risk of myopia.

## 5 Potential biological mechanisms between inflammation and myopia

Overall, the potential biological mechanisms by which inflammation affects myopia may include the direct induction of scleral remodeling by inflammatory signaling pathways, including MAPK and NF-κB, and the indirect effects of the influence of inflammation on retinal and choroidal blood vasculature, interference with dopamine, modulation by extracellular vesicles (EVs) and regulation of the refractive index of the lens.

### 5.1 Direct contribution of inflammatory signaling pathways MAPK and NF-κB to scleral remodeling

From an anatomical and pathological point of view, the sclera, in common with cartilage, tendons, bone, ligaments, dermis, and perivascular muscle tissue, is of dual neural crest and mesodermal tissue origin and maintains the potential to form cartilage throughout evolution (77). Thus, similarly to articular cartilage, the sclera is often a target of inflammatory cells in immunoinflammatory diseases (78). From this perspective, a correlation between inflammation and myopia is possible.

**Figure 1:**
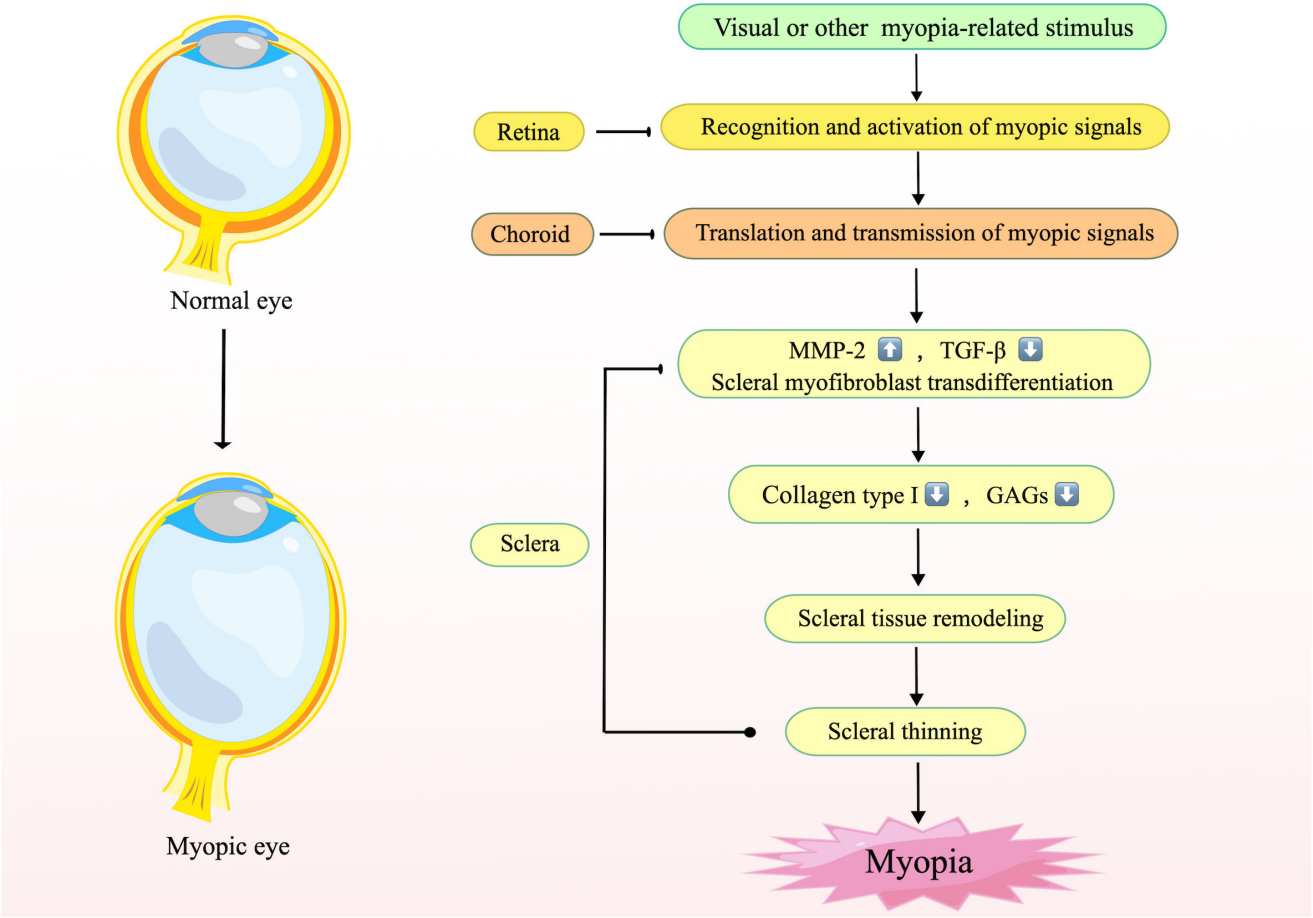
The mechanism of scleral remodeling during myopia. This figure illustrates the general mechanism of scleral remodeling in myopia. The retina recognizes stimuli or visual signals associated with myopia and transmits them to the sclera via the choroid, resulting in elevated MMP-2, reduced TGF-β, and transdifferentiation of scleral myofibroblasts, which leads to changes in the composition of the scleral stroma, elongation of the posterior pole of the eye, and finally the development of myopia. Created by figdraw.com.

In terms of molecular mechanisms and signaling pathways, myopigenesis may initiate in the retina, pass through the choroid, and finally reach the sclera, inducing scleral remodeling (79). During this process, the retina-sclera signaling cascade promotes the increase of MMP-2, the degradation of TGF-β, and scleral myofibroblast transdifferentiation, resulting in abnormal extracellular matrix (ECM) metabolism, reduction of collagen type I and glycosaminoglycans (GAGs). Subsequently, the sclera undergoes tissue remodeling and thinning of the posterior pole, leading to the formation of axial myopia (80) (**Figure 1**). Inflammation attracts cytokines, blood cells, growth factors and so on to the site of infection or injury, then allows tissue remodeling through protein hydrolytic activity and function or connective tissue rebuilding (81). Therefore, given the inflammatory susceptibility of the sclera and the pathophysiological mechanisms promoted by inflammation, it can be assumed that the direct contribution of inflammation to myopia onset may be accomplished through a retina-sclera signaling cascade that induces scleral remodeling.

TNF-α is a proinflammatory cytokine produced mainly by innate immune cells or T cells that exerts multiple biological activities by binding and activating two different receptors (82). Ligation of TNF-α with TNF receptor 1 leads to the assembly of complex I, which then activates NF-κB and MAPKs to promote inflammation and tissue degeneration (83). Activation of TNF receptor 2 also results in the assembly of complex I and activation of downstream signaling pathways which mainly mediates homeostatic bioactivities (83). Ocular TNF-α is mainly released by microglia and Müller cells, causing apoptosis of retinal pigment epithelial cells and disrupting the blood−retinal barrier through activation of the epidermal growth factor receptor (EGFR)/p38/NF-κB/p62 pathway (84). Additionally, it can positively stimulate glial proliferation and accelerate the release of other proinflammatory factors of Müller cells (85).

As a pleiotropic cytokine that promotes inflammation, IL-1β is rarely present in cells of healthy individuals. Its transcription is induced by TNF-α, IL-18, IL-1α or IL-1β itself through activation of the NF-κB pathway (86). Mature IL-1β activates TGF-β-activated kinase 1(TAK1) through a series of signaling pathways, which then initiates the MAPK cascade response and NF-κB transcription (87), promoting inflammatory mediator recruitment such as IL-6, IL-8 and TNF-α and local/systemic inflammatory responses. Increased ocular IL-1β is capable of triggering an immediate inflammatory response in the retina, destroying retinal capillary endothelial cells, inducing angiogenesis and causing dysregulation of nitric oxide (NO) (88), the expression of which was increased during FDM (89).

IL-6 is a cytokine with pro- and anti-inflammatory properties that is activated by innate immune cells during inflammation. The classical pathway of IL-6 bound to the IL-6 receptor exerts anti-inflammatory properties, whereas trans-signaling of IL-6 bound to the soluble IL-6 receptor acts as a pro-inflammatory mediator (90). It activates MAPKs and NF-κB through activation of Janus kinase (JAK) to mediate downstream responses (91). Trans-signaling of IL-6 can lead to oxidative stress, endothelial cell dysfunction, inflammation and neovascularization within the human retina (92). Dysregulated and persistent production of IL-6 has been implicated in the development of various chronic inflammatory diseases (93)including inflammatory ocular disease (94).

MMPs are key enzymes involved in ECM remodeling. MMP-2 expression preceding myopigenesis can be observed in FDM, whereas its decline can also directly impede myopia progression (95). The expression of MMPs is upregulated at the transcriptional level by many inflammatory cytokines (96). Activated MMPs are able to regulate the availability and activity of inflammatory mediators such as TNF-α and IL-1β and induce the migration of inflammatory cells to inflammatory sites by modifying chemotactic agents (97).

MAPK, a kind of evolutionarily highly conserved serine/threonine protein kinase, contains several members, including c-Jun NH2-terminal kinase (JNK), p38 MAPK, and extracellular signal-regulated kinase (ERK) (98). Inflammatory cytokines or other stimuli trigger signal transduction by sequentially activating MAP kinase kinase kinase (MAPKKK), MAP kinase kinase (MAPKK), and MAPK, and then activate downstream kinases or transcription factors to mediate cell proliferation, differentiation, apoptosis, and inflammatory responses (99). As a transcription factor, NF-κB is a central mediator of proinflammatory gene induction and functions in both innate and adaptive immune cells, which can induce the production of downstream molecules such as inflammatory cytokines, chemokines, and adhesion molecules, directly targeting inflammation (100). In resting status, the majority of NF-κB consisting of p50 and p65 heterodimers remains inactive by bounding to the inhibitory proteins of NF-κB (IκB) in the cytoplasm. Upon receipt of activation signals, the activated and phosphorylated IκB kinase (IKK) complex, including catalytic subunit IKKα, IKKβ and regulatory subunit IKKγ (also known as NF-κB essential modulator, NEMO), leads to the ubiquitination and degradation of IκB. The degraded IκB thereby releases activated NF-κB dimers into the nucleus and subsequently regulates gene transcription (101). There are also interactions existing between the MAPK and NF-κB signaling pathways. Phosphorylation of IκB can be activated by activated protein kinases downstream of MAPK, and similarly, NF-κB can mediate the activation of downstream targets of MAPK on inflammatory cytokines (102). Both pathways function in ocular surface inflammation, such as dry eye, keratitis, and allergic conjunctivitis, and regulate apoptosis of retinal ganglion cells (103, 104).

**Figure 2:**
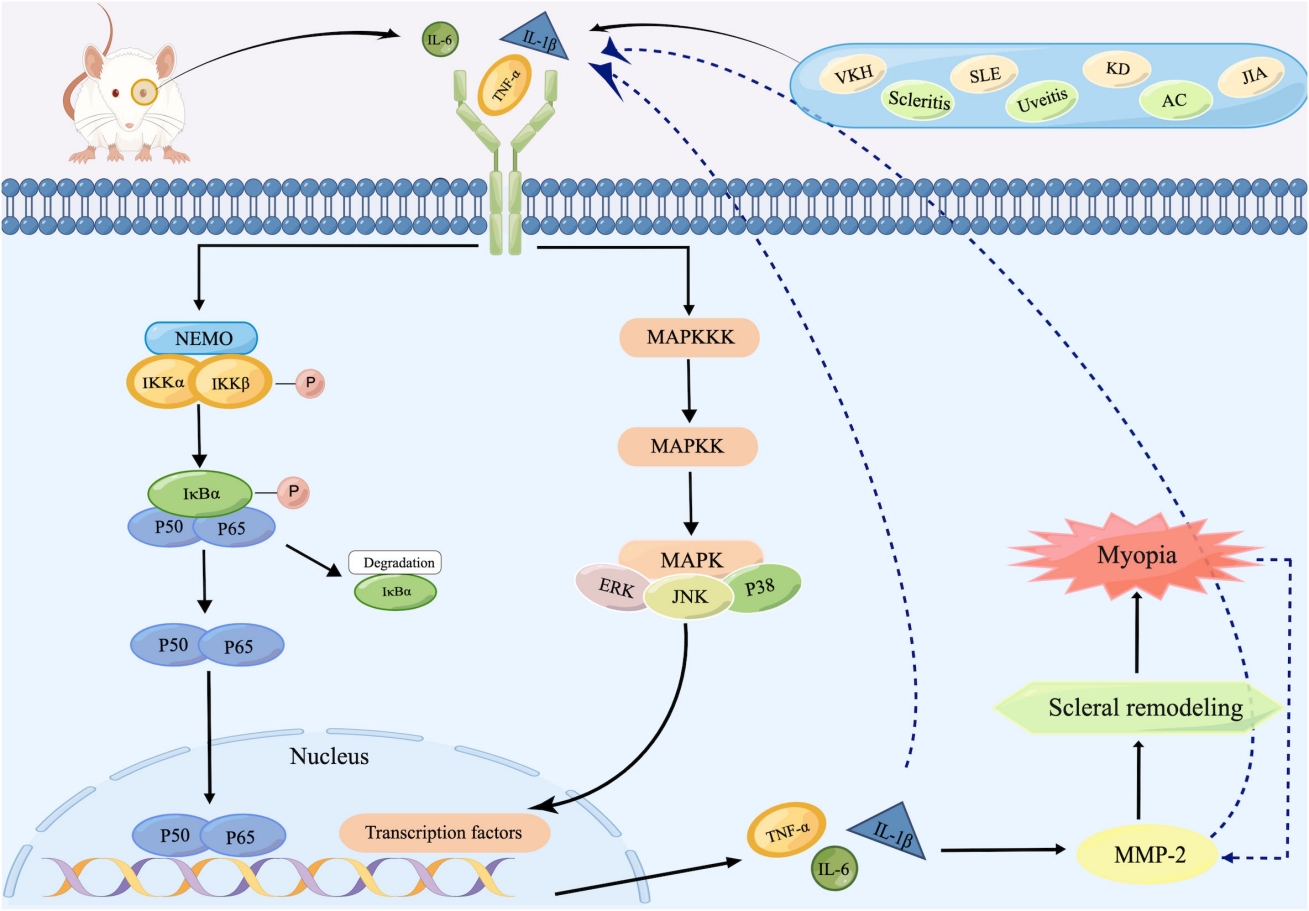
Schematic representation of inflammatory signaling pathways inducing scleral remodeling. After recognizing inflammatory stimuli, increased inflammatory cytokines such as TNF-α, IL-1β, and IL-6 in the retina activate IKK as well as MAPKKK, initiating NF-κB as well as MAPK signaling pathways. Both two ultimately trigger the expression of TNF-α, IL-1β, and IL-6, which then activate MMP-2 and subsequently lead to scleral remodeling and finally the onset of myopia. During this process, the increased MMP-2 produced by progressive myopia re-activates TNF-α, IL-1β, and IL-6, whose increase act as inflammatory stimulus for the reactivation of both pathways. Created by figdraw.com.

Given the considerable overlap in the target genes activated by the MAPK and NF-κB signaling pathways, it is hypothesized that both signaling pathways may be jointly involved in the pathological process of myopia (**Figure 2**). Ocular inflammatory diseases or abnormal visual stimulus in the retina stimulate the production of inflammatory cytokines, such as TNF-α, IL-1β, and IL-6. These increased cytokines then activate MAPKKK and IKK through a series of signal transduction, and consequently trigger MAPK and NF-κB signaling pathways, which initiate downstream signaling and drive the production of numerous proinflammatory cytokines. These agents then activate MMP-2 expression in the retina, followed by the sclera, leading to cleavage of collagen, causing scleral remodeling as well as the onset of myopia. In this process, activated TNF-α and IL-1β also promote the expression of other proinflammatory factors, such as IL-6, IL-8, and MCP-1, while acting again on MAPKKK as well as IκB (101) to regulate both pathways. Meanwhile, the progression of myopia produces more MMP-2, promoting further release of inflammatory factors and ultimately creating a malignant cycle.

### 5.2 Impact of inflammation on the retinal and choroidal vasculature

Increased ocular inflammation may also affect myopia through the ocular vasculature. Chronic inflammation activates endothelial cells, and endothelial dysfunction leads to reduced vasodilator function, leukocyte recruitment, decreased NO bioavailability, and increased oxidative stress (105), triggering impaired ocular microvascular circulation and oxygen supply. Since retinal and choroidal vascular microcirculation provides oxygen and material exchange directly to the retina and sclera (106), the impaired ocular microvascular circulation and increased oxidative stress caused by inflammation may directly lead to inadequate perfusion and reduced supply of oxygen. The scleral hypoxia causes the accumulation of HIF-1α, myofibroblast transdifferentiation, and decreased collagen production, ultimately leading to scleral remodeling and the development of myopia (107). This is consistent with the reduced density and lower blood flow in the choroidal and retinal microvasculature and reduced oxygen saturation in small retinal arteries that occur in myopic eyes (108, 109). Additionally, with obstruction of blood flow, monocytes within the choroidal vessels gradually migrate toward the sclera due to increased vascular permeability and subsequently differentiate into MMP-2 (45), again aggravating myopia.

### 5.3 Inflammatory interference with dopamine

As an important factor in myopic regulatory pathways, the protective effect of dopamine on myopia has been well documented (7). Available evidence also suggests that inflammatory cytokines can potentially affect multiple aspects of dopamine neurotransmission, leading to decreased synthesis, impaired packaging or release, and increased reuptake of dopamine, ultimately resulting in reduced dopamine signaling in the basal ganglia (110). Therefore, it can be speculated that increased inflammatory cytokines in the eye may similarly reduce ocular dopamine by interfering with dopamine synthesis and release in retina to exacerbate myopia development.

### 5.4 Inflammatory modulation by extracellular vesicles

In view of the important role of EVs in intercellular communication and inflammatory regulation, they may serve as an additional bridge between inflammation and myopia. EVs, including microvesicles, exosomes, and apoptotic bodies (111), are capable of carrying numerous cytokines, such as IL-6, TNF-α, CCL2, and TGFβ, protecting them from enzymatic degradation and delivering them to distant cells (112). All types of immune cell involved in inflammation can secrete EVs, while EVs are able to influence the behavior of innate immune cells and cytokine expression by transferring various mediators to modulate the level of inflammation (113). In pathological states, ocular exosomes could mediate ECM remodeling, retinal inflammation, and blood-retinal barrier dysfunction (114). Lately, researchers found that exosomes in the aqueous humor of myopic patients contained more total RNA compared to control group (115), and key exosomal microRNAs, such as miR-143-3p, miR-145-5p and has-miR-518d-3p associated with high myopia and pathological myopia were successively identified (115–117), suggesting that exosomes also contribute to the process of myopia. Due to the small particle size and well traversed ability to the blood-retinal barrier (BRB) (118), it is reasonable to speculate that in systemic inflammatory diseases, elevated inflammatory cytokines in the peripheral blood may be carried by EVs and enter the eye through the BRB, thereby affecting myopia. Meanwhile, ocular exosomes may induce an exacerbation of ocular inflammatory state through activation of immune cells in the eye, hence participating in the development of myopia.

### 5.5 Regulation of the refractive index of the lens by inflammation

In patients with certain inflammatory or immune diseases, apart from the possible mechanisms described previously, increased inflammation may also cause reversible or permanent myopia through uveal effusion or ciliary swelling, which may relax the suspensory ligament and ciliary muscle, increasing the distance between the fovea and lens and subsequently increasing the refractive index of the lens.

## 6 Potential of anti-inflammatory drug to intervene myopia

The main measures considered to reduce the occurrence of myopia and prevent its progression include public health interventions, optical measures, and pharmacological treatments. Among these, spending more time outdoors can significantly reduce the prevalence of myopia (119); multifocal soft contact lenses, peripheral plus spectacles, multifocal spectacles, and orthokeratology have been shown to slow refractive change and axial elongation (120). For pharmacological measures, the daily application of low-dose atropine eye drops has been widely used as medical prophylaxis for myopia progression (121). However, atropine has side effects such as rebound after discontinuation, hotophobia or glare, blurred vision (particularly for near vision) and hypersensitivity reactions (120). Finding more drugs for the treatment of myopia based on promising signaling pathways is one of the current research hotspots.

The hypothesis that myopia may be suppressed by the anti-inflammatory effects of drugs is supported by the association between inflammation and myopia. Lactoferrin (122), diacerein (36), and resveratrol (37) have been shown to reduce the progression of experimental myopia in animals by inhibiting the expression of inflammatory cytokines related to the MAPK or NF-κB signaling pathways in the retina. The mechanism of atropine treatment of myopia also involves inhibition of muscarinic acetylcholine receptor activation and consequent inhibition of inflammation-related signaling pathways (18).

Besides, given that artificial exosomes have been shown to be able to treat ocular inflammatory diseases by inhibiting the infiltration of inflammatory cells (123), reducing the expression of pro-inflammatory cytokines (124), and protecting the structure and function of the retina and retinal ganglion cells (125), it is reasonable to believe that artificial exosomes may also be a new target for myopia treatment.

Therefore, anti-inflammatory drugs and artificial exosomes may become a new option for the treatment of myopia after low-dose atropine. However, as the relationship and exact mechanism between inflammation and myopia are not yet defined, the efficacy has only been confirmed in animal experiments, and the safety, dose, mode of administration, and side effects of these drugs are still unknown. Further verification is needed to determine whether anti-inflammatory drugs can actually be used to treat myopia in humans.

## 7 Summary and future

**Figure 3:**
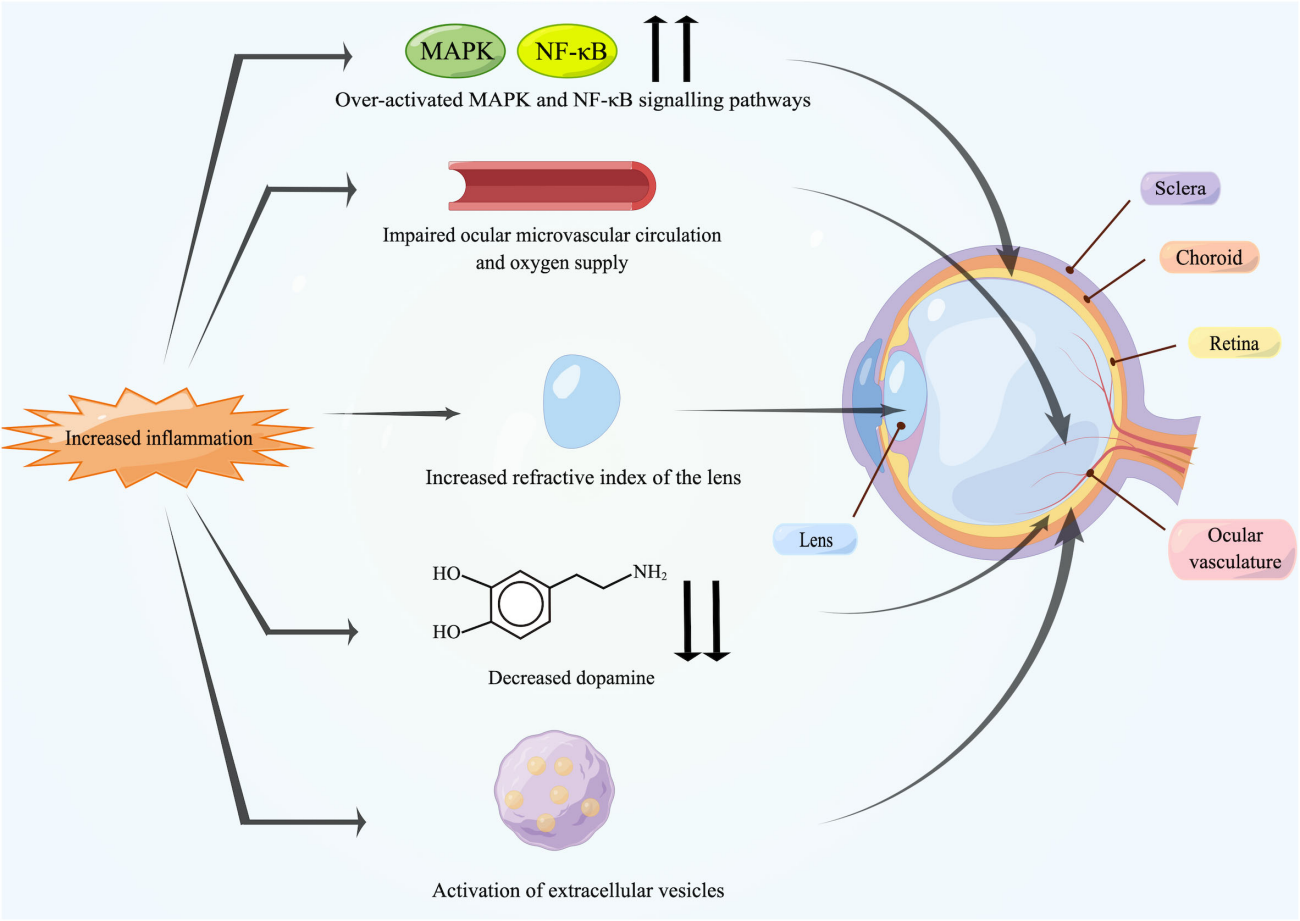
Potential mechanisms between inflammation and myopia. Increased inflammation can induce myopia. The over-activated MAPK and NF-κB signaling pathways activate inflammatory factors and MMP-2 in the retina, followed by transmission to the sclera, leading directly to scleral remodeling. Chronic inflammation damages the endothelium of ocular vasculature, causing impaired blood circulation and triggering ischemia of retina and choroid as well as scleral hypoxia. Meanwhile, inflammation also leads to the reduction in retinal dopamine release and the activation of EVs. In addition, some patients may suffer from uveal effusion or ciliary swelling by inflammation, which ultimately leads to increased refractive power of lens. Created by figdraw.com.

To our knowledge, this is the first review to comprehensively interpret the association between myopia and inflammation. Low-grade inflammation in the body can induce myopia progression, and the prevalence of myopia has been found to be elevated in patients with inflammatory or immune diseases. The association between them has also been confirmed in experimental myopia. In addition, anti-inflammatory drugs and artificial exosomes have inhibitory effects on myopia. Specific mechanisms of inflammation-induced myopia may include scleral remodeling caused by dysregulation of the MAPK and NF-κB signaling pathways and the effects of inflammation on the ocular vasculature, dopamine, the inflammatory modulation by EVs and the refractive index of the lens (**Figure 3**).

Considering the impact of inflammation on myopia, more frequent ophthalmic screening, more outdoor activities and education are needed to prevent myopia in younger patients with inflammatory or immune diseases, while stronger interventions are needed in those who are already myopic. Additionally, given the effectiveness of anti-inflammatory drugs in animal studies, the possibility of combining anti-inflammatory products with low concentrations of atropine to better control myopic progression could be considered. Furthermore, in adult patients with sudden myopia progression, it is important to be alert for insidious onset of endophthalmitis, such as uveitis, posterior pole scleritis, and choroiditis.

In conclusion, although the association between inflammation and myopia has not been fully assessed, inflammation may be a potential new target for myopia. As potential regulators of inflammation, macrophages, microglia, and endothelial cells in the eye may be a promising area of research, especially with regard to the precise regulation of immune cells, the timing of activation, and cell-cell communication. Meanwhile, investigations on the exact causal relationship between inflammation and myopia, the molecule signaling pathways in myopia, the therapeutic effects of artificial exosomes and anti-inflammatory drugs should not be neglected.

## Author contributions

RX: Writing – original draft. JZ: Writing – original draft. LL: Writing – review & editing. WZ: Writing – review & editing.
